# *Plasmodium knowlesi *in humans, macaques and mosquitoes in peninsular Malaysia

**DOI:** 10.1186/1756-3305-1-26

**Published:** 2008-08-19

**Authors:** Indra Vythilingam, Yusuf M NoorAzian, Tan Cheong Huat, Adela Ida Jiram, Yusof M Yusri, Abdul H Azahari, Ismail NorParina, Abdullah NoorRain, Sulaiman LokmanHakim

**Affiliations:** 1 Parasitology Unit, Infectious Disease Research Centre, Institute for Medical Research, Jalan Pahang, 50588, Kuala Lumpur, Malaysia; 2 Environmental Health Institute, The National Environment Agency, Singapore; 3 Entomology Unit, Infectious Disease Research Centre, Institute for Medical Research, Jalan Pahang, 50588, Kuala Lumpur, Malaysia; 4 Bioassay Unit, Herbal Medicine Centre, Institute for Medical Research, Jalan Pahang, 50588, Kuala Lumpur, Malaysia; 5 Infectious Disease Research Centre, Institute for Medical Research, Jalan Pahang, 50588, Kuala Lumpur, Malaysia

## Abstract

**Background:**

Since a large focus of human infection with *Plasmodium knowlesi*, a simian malaria parasite naturally found in long-tailed and pig tailed macaques, was reported in Sarawak, Malaysian Borneo, it was pertinent to study the situation in peninsular Malaysia. A study was thus initiated to screen human cases of *Plasmodium malariae *using molecular techniques, to determine the presence of *P. knowlesi *in non- human primates and to elucidate its vectors.

**Methods:**

Nested polymerase chain reaction (PCR) was used to identify all *Plasmodium *species present in the human blood samples sent to the Parasitology laboratory of Institute for Medical Research. At the same time, non-human primates were also screened for malaria parasites and nested PCR was carried out to determine the presence of *P. knowlesi*. Mosquitoes were collected from Pahang by human landing collection and monkey-baited-traps situated on three different levels. All mosquitoes were identified and salivary glands and midguts of anopheline mosquitoes were dissected to determine the presence of malaria parasites and nested PCR was carried out on positive glands. Sequencing of the csp genes were carried on *P. knowlesi *samples from humans, monkeys and mosquitoes, positive by PCR.

**Results and Discussion:**

*Plasmodium knowlesi *was detected in 77 (69.37%) of the 111 human samples, 10 (6.90%) of the 145 monkey blood and in 2 (1.7%) *Anopheles cracens*. Sequence of the csp gene clustered with other *P. knowlesi *isolates.

**Conclusion:**

Human infection with *Plasmodium knowlesi *is occurring in most states of peninsular Malaysia. *An. cracens *is the main vector. Economic exploitation of the forest is perhaps bringing monkeys, mosquitoes and humans into increased contact. A single bite from a mosquito infected with *P. knowlesi *is sufficient to introduce the parasite to humans. Thus, this zoonotic transmission has to be considered in the future planning of malaria control.

## Background

The burden of malaria in many parts of peninsular Malaysia has decreased substantially due to malaria control activities. The reported incidence of malaria in Malaysia has decreased to 5,294 cases in 2006 compared to 12,705 cases in 2000. In 2006 there were only 852 cases in peninsular Malaysia compared with 3918 cases in 2000 (Annual Report, Ministry of Health Malaysia). However, a fifth species, *Plasmodium knowlesi *that was originally described as a malaria parasite of the long-tailed macaque monkeys [1] is now occurring here. The first case was reported in peninsular Malaysia in 1965 [2]. However, since 2004 there have been reports of P. *knowlesi *infecting humans in the Southeast Asia region [3-8].

The accidental discovery that *Plasmodium cynomolgi *could be transmitted to humans *via *mosquito bites in the laboratory [9] stimulated great interest and thus, extensive studies were carried out in peninsular Malaysia to determine the distribution, prevalence and species of malaria parasites in monkeys and apes and the vectors of monkey malaria in nature and to determine whether monkey malaria infection transmissible to man existed in Malaya [10-14]. From their studies, several new species of simian malaria parasites were described [15-17].

After the first human case of *P. knowlesi*, a study that was initiated in the state of Pahang to investigate whether malaria was a zoonosis, concluded that simian malaria in humans was an extremely rare event [18,19]. This was based on studies in which blood samples were collected from more than 1100 local residents, the samples were pooled and injected them into rhesus monkeys. However, none of the monkeys contracted malaria.

In most instances, infection of the human host with the malaria parasite begins with the bite of an infected *Anopheles *mosquito that inoculates sporozoites into the host. In the early works carried out on the vectors of simian malaria in peninsular Malaysia, it was postulated that *Anopheles leucosphyrus *complex are involved in simian malaria transmission. In a study conducted in the coastal area of Selangor in peninsular Malaysia, *Anopheles hackeri *was incriminated as a vector of *P*.*knowlesi *[10]. In the inland hill forest area of Selangor, (Hlu Lui) a simian parasite, *Plasmodium inui *was isolated from *Anopheles latens *(= *An. leucosphyrus*) [11]. A year later in 1963 *Plasmodium cynomolgi *was isolated from *Anopheles introlatus *(= *An. balabacensis introlatus*) [12]. In the monsoon rain forest of northern Malaysia in the state of Perlis *An*. *cracens *(= *An. balabacensis balabacensis*) was the vector of both *P. inui *and *P. cynomolgi *[13].

In 2004, a large focus of human *P. knowlesi *infection was reported in the Kapit Division of Sarawak [3]. Thus, it was pertinent to deduce if knowlesi malaria was currently occurring in peninsular Malaysia and to elucidate the vectors and to study the parasites in macaques in the areas around human cases. This paper reports the preliminary results of the study.

## Methods

### Human blood samples

Blood samples or Giemsa stained blood films were sent to the Parasitology Unit of the Institute from hospitals and health centres that wanted a confirmation of *Plasmodium malariae *or in some cases to rule out *P. knowlesi*.

### DNA extraction from whole blood

DNA was extracted from whole blood using the Qiagen D Neasy Blood Tissue Kit (Hilden, Germany), following the manufacturer's recommendations.

### DNA extraction from blood films

In some cases only Giemsa stained blood films were provided. Before the extraction of the DNA, the slides were first cleaned with chloroform to remove oil. Fifty microliters of TE buffer was then pipetted on to the thin blood film. Two discs were punched out from a Whatman 1 filter paper (Whatman USA) using a pre flamed paper puncher. The discs were placed on the slide to soak up the buffer. Using a clean, flamed forceps, at least half of the smear was completely wiped off the slide with the filter paper and was transferred to 1.5 ml centrifuge tubes (Axygen). The DNA was extracted using the Qiagen D Neasy Blood Tissue Kit (Hilden, Germany).

### Nested Polymerase Chain Reaction

Nested PCR assays [3,20], based on the *Plasmodium *DNA sequence of the small subunit ribosomal RNA (SSUrRNA) genes, were used to detect and identify the species of malaria parasites found in the blood. The maximum number of samples processed at any one time was not more than 10. Positive controls for *P. falciparum*, *P. knowlesi, P. malariae *and *P. vivax *were included for all nested PCR species assays. A negative control was also included for each batch of assays. Nest 1 reaction was carried out in a 50 μl reaction mixture containing 1× reaction buffer (5× Green Go Taq Flexi Buffer, Promega Madison USA) 3 mM MgCl_2_(Promega), 200 mM of each deoxynucleoside triphosphate (Promega), 300 nM of each primers and 1.25 U of Go Taq DNA polymerase (Promega) and 5 μl of DNA template was used for each reaction.

Nest 2 PCR amplification was done in a 20 μl reaction mixture containing 1× reaction buffer (5× Green Go Taq Flexi Buffer Promega) 2 mM MgCl_2_(Promega,), 200 mM of each deoxynucleoside triphosphate (Promega,), 300 nM of each primers and 0.5 U Go Taq DNA polymerase (Promega,) and 2 μl of the nest 1 PCR products were used as DNA templates. All PCR reactions were carried out using thermal cycler (Techne TC 152 -Barloworld Sci Ltd UK). Ten microliters of the nest 2 amplicons were analyzed by agarose gel.

### Sequencing of the Plasmodium csp genes

The *csp *genes of malaria parasites from all human isolates positive for *P. knowlesi *were amplified with primers, PKCSP-F and PKCSP-R [3]. However, for the mosquito and monkey isolates, primers PKCSPF2 (5' TACAAGAACAAGATGARGAAC 3') and PKCSPR2 (5' TCAGCTACTTAATTGAATAATGC 3') were used since many non- specific bands were obtained with PKCSP-F and PKCSP-R.

PCR was carried out in a 20 μl reaction volume containing 1× Phusion HF buffer (Finnzymes), 200 mM/l of each dNTP (Finnzymes Finland) and 250 nM/l of each primer, and 0.02 U of Phusion DNA Polymerase (Finnzymes Finland). The PCR was carried out using a thermal cycler (Techne TC 152 -Barloworld Sci Ltd UK). The PCR conditions were as follows: initial denaturation at 98°C for 30 sec followed by 40 cycles of amplification at 94°C for 7 sec, 51°C for 20 sec, 72°C for 20 sec followed by a final extension step of 10 min. The expected size of the PCR products is approximately 1.2 kb and amplicons from each isolate were excised from the gel and purified using Perfectprep gel cleanup kit (Eppendorf, Germany), following the manufacturer's recommendation. The purified products were cloned as previously described [3]. At least 20 of transformants from each PCR were screened using the *csp *primers mentioned above. Amplification was done in a 20 μl reaction mixtures containing 1× reaction buffer (5× Green Go Taq Flexi Buffer, Promega Madison USA) 2 mM MgCl_2_(Promega), 200 mM of each deoxynucleoside triphosphate (Promega), 300 nM of each primers and 0.5 U Go Taq DNA polymerase (Promega,). PCR conditions were as follows: initial denaturation of 94°C for 10 min followed by 30 cycles of amplification at 94°C at 1 min, annealing at 53°C at 1 min, extension at 72°C for 1 min 20 sec, followed by a final extension step at 72°C at 5 min. Ten microliters of the amplicons were digested with *Eco*R1 (Promega) and analyzed by gel electrophoresis. Plasmids from clones having the correct inserts were extracted using S.N.A.P. plasmid extraction kit (Invitrogen, USA) following the manufacturer's protocol. Purified plasmids was sent to Solgent Company Limited (Daejeon, South Korea) for sequencing to obtain the entire *csp *gene sequence of *P. knowlesi*

### Analysis of sequence data

Analysis of the csp genes was performed as previously described [3]. Sequences of the 456 nucleotide that encodes non – repeat N- terminal (first 195 nucleotides of coding sequence) and C- terminal (the last 261 nucleotides of the *csp *gene coding sequence) region of the protein were aligned by CLUSTAL W using Megalign (Lasergene, DNASTAR, USA). Regions of the protein were aligned using Megalign software (Lasergene). The *csp *gene sequences from patients, mosquitoes and monkey samples were compared with those obtained from the GenBank data base. Phylogenetic trees were performed by the neighbour-joining (NJ) [21] and Bayesian methods [22]. The NJ method was analyzed using the Kimura-2 parameter with 1000 bootstrap replicates and was carried out using the MEGA version 4.0 software [23]. On the other hand, the Bayesian method was analysed using the Hasegawa-Kishino-Yano (HKY) model with the following parameters: the search was performed at 1,000,000 generations, sampling every 100 generations and the first 2000 trees were discarded in the burn-ins. The analysis was carried using the Mr. Bayes 3.1 software [24].

Nucleotide sequences reported in this study have been deposited in Gen Bank under accession numbers: EU687467–EU687470, EU708437 (human samples), EU821335 (mosquito sample), EU821336 (monkey sample). The other malaria *csp *gene sequences used were obtained from GenBank: *P. knowlesi *(M11031), *P. knowlesi *(K0082), KH35 (AH013332), KH43 (AH013333), KH50 (AH013334), KH107 (AH013336), KH115 (AH013337), *P. coatneyi *(AY135360), *P. cynomolgi *(M15104), *P. simiovale *(U09765), *P. simium *(L05068), *P. inui *(FJ009512) *P. vivax *(M34697), *P. malariae *(U09766), *P. malariae *(J03992), *P. falciparum *(K02194) and *P. vinckei lentum *(AF162331).

### Trapping of monkeys

Monkeys were trapped in the vicinity of Kuala Lumpur, Selangor State and Kuala Lipis in Pahang State with help from the wild-life department. The captured monkeys were anesthetized by intramuscular injection with ketamine hydrochloride. Ten ml blood was collected, after which the monkeys were tagged with an electronic identification system (Trovan Ltd London). After they recovered they were released into the deep forest.

### Preparation of blood films

Both thick and thin blood films were prepared followed by staining with Giemsa. Microscopic examinations were carried out using compound microscope under oil immersion under 100× magnification.

### DNA extraction from whole blood, PCR and sequencing

DNA was extracted from the whole blood using the Dneasy Tissue Extraction Kit (Qiagen, Germany) following the manufacturer's recommendation. All monkeys were screened for malaria parasites using primers rPLU3 and rPLU4 [20], and for *P. knowlesi *using the primers Pmk8 and Pmk9 [3]. Nested PCR, cloning and sequencing of all samples positive for *P. knowlesi *were performed as described above.

### Study Sites for mosquito collection

The study site for mosquito collection was in Kuala Lipis district in the State of Pahang. Pre-surveys for mosquito collections were carried out to determine suitable sites for long term study based on presence of monkeys and occurrence of cases. Based, on pre-surveys two sites were selected for the study. One is Serunai Mela village [4° 7.0'N, 102° 11.9'E ] and the other is a fruit farm in Sungai Ular [4° 15.7'N, 102°4.8'E ]. In Serunai Mela, a case of *P. knowlesi *had occurred and the case house is situated at the forest fringe. Sungai Ular was selected as a patient had reported that he visited the farm before falling ill and it was also frequented by monkeys

#### Mosquito collections

All night mosquito collections using bare-leg catch method [25] were performed from July 2007 to November 2007. In each area 4 nights of collection were carried out every month by three men working outdoors from 18.00 to 06.00 hours.

#### Monkey-baited-trap

In order to compare the number of mosquitoes attracted to humans and monkeys, a monkey- baited – trap was constructed in Serunai Mela Village as previously described [26,27].

### Mosquito identification and dissection

All mosquitoes were identified morphologically in the field laboratory. The keys of Reid [28] were used for the identification of *Anopheles *mosquitoes and keys of Sallum [29] were used for *leucosphyrus *group in particular. *Anopheles *mosquitoes were dissected to extract ovaries to determine parity and the midguts and salivary glands were examined for oocysts and sporozoites, respectively. All the positive salivary glands were placed in 1.5 microcentrifuge tubes (Axygen, USA) containing absolute alcohol and were labeled accordingly.

### DNA extraction, PCR and sequencing

Ethanol used in the preservation of the salivary glands were allowed to evaporate completely by placing the tubes in a Thermomixer (Eppendorf, Germany) set at 70°C. DNA was then extracted using the Qiagen D Neasy Blood Tissue Kit (Hilden, Germany) as described above. PCR and sequencing were also carried out as mentioned above.

### Ethical clearance

This project was approved by the Institute for Medical Research & Ethical Committee Ministry of Health Malaysia and the Animal Use Committee of the Institute.

## Results

### Humans

**Figure 1:**
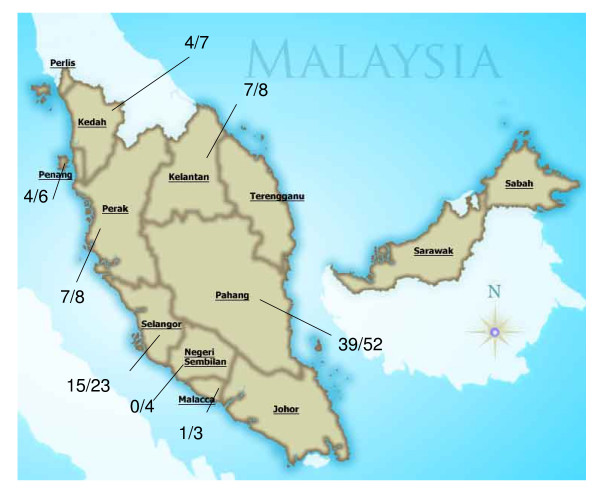
**Map of Malaysia showing cases of *P. knowlesi* by PCR in P. Malaysia.** Denominator indicates the total number of samples for each state.

A total of 111 samples were received for PCR (from July 2005–March 2008). Of these 77 (69.37%) were positive for *P. knowlesi *(Table 1). By microscopy, 93 (83.78%) of the slides were reported as *P. malariae *of which by nested PCR 62 (55.86%) were positive for *P. knowlesi *and 11 (9.91%) were mixed infection with *P. knowlesi *and other human malaria parasites (Table 1). Positive *P. knowlesi *cases were observed in all states in peninsular Malaysia with the exception of four – Johore, Negeri Sembilan, Perlis and Terengganu (Fig. 1). Pahang has the highest number of *P. knowlesi *cases (50.65%).

**Table 1:** Results of blood samples of malaria obtained by microscopy and PCR

	Cases detected by microscopy					
		
PCR results	Pf	Pv	Pm	Pf+Pm	Pm+Pv	Cases detected by PCR
Pf	4	1	1			6
Pv		6	3	1	1	11
Pm			16			16
Pk	2	1	62			65
Pf+Pm			1			1
Pf+Pk			2			2
Pv+Pk		1	5		1	7
Pk+Pm			3			3
Total	6	9	93	1	2	111

Pf = *Plasmodium falciparum*; Pv = *Plasmodium vivax*, Pm = *Plasmodium malariae*, Pk = *Plasmodium knowlesi*

### Monkeys

Until December 2007 a total of 145 monkeys had been trapped. Of these 143 (98.62%) were *Macaca fascicularis *and one (0.69%) each of *M. nemestrina *and *Presbytis melalophos*. Seventy five of the monkeys were trapped from Kuala Lipis and of these 73 (97.33%) were positive for malaria parasites by microscopy and 10 were positive for *P. knowlesi *by PCR. In Kuala Lumpur, 2 (6.90%)were positive for malaria parasites out of the 29 that were trapped but none was positive for *P. knowlesi*. In Selangor, all 41 monkeys examined were negative.

### Mosquitoes

A total of 339 *Anopheles *mosquitoes belonging to 12 species were caught biting humans and monkeys in the five months period as shown in Table 2. *Anopheles cracens *was the predominant mosquito comprising 62.2% of the total collection. This was followed by *Anopheles maculatus*. The two species attracted to monkeys were *An. cracens *and *Anopheles kochi*.

**Table 2:** Mosquitoes collected from two sites in Kuala Lipis, Pahang from July to November 2007.

*Anopheles *species	Serunai Mela		Sg Ular	Total (%)
	
	BLC	MBT		
*An. aconitus*	0	0	4	4 (1.2)
*An. barbirostris *gr	2	2	2	6 (1.8)
*An. cracens*	107	19	85	211 (62.2)
*An. hyrcanus *gr	8	1	0	9 (2.7)
*An. kochi*	1	15	0	16 (4.7)
*An. maculatus*	4	0	67	71 (20.9)
*An. phillippinensis*	3	0	3	6 (1.8)
*An. pujutensis*	0	1	0	1 (0.3)
*An. separatus*	0	0	5	5 (1.5)
*An. tessellatus*	4	0	3	7 (2.1)
*An. umbrosus*	1	0	0	1 (0.3)
*An. vagus*	1	1	0	2 (0.6)

Total	131	39	169	339

### Sporozoite rate

A total of two *An. cracens *were positive for sporozoites from Serunai Mela of which one was positive for oocyst as well. Thus, the sporozoite rate in Serunai Mela was 1.7. PCR results found them to be positive for *P. knowlesi *and this was confirmed by the sequencing of the CSP gene.

### Biting cycles

*Anopheles cracens *were early biters coming to bite man as early as 19.00 hours and the peak biting time was 19.00 to 21.00 hours. In the forest, the biting was reduced after 22.00 hours but in the fruit orchard they continued to bite throughout the night.

### Sequencing analysis of the *csp *genes

**Figure 2:**
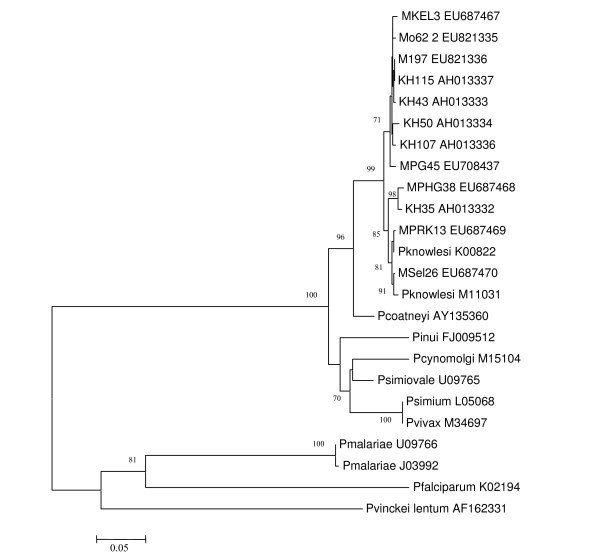
**Phylogenetic tree based on the non-repeat region of the circumsporozoite (*csp*) genes of malaria parasites produced by the neighbor-joining method.** Figures on the branches are bootstrap percentages based on 1000 replicates and only those 70 and above shown.

**Figure 3:**
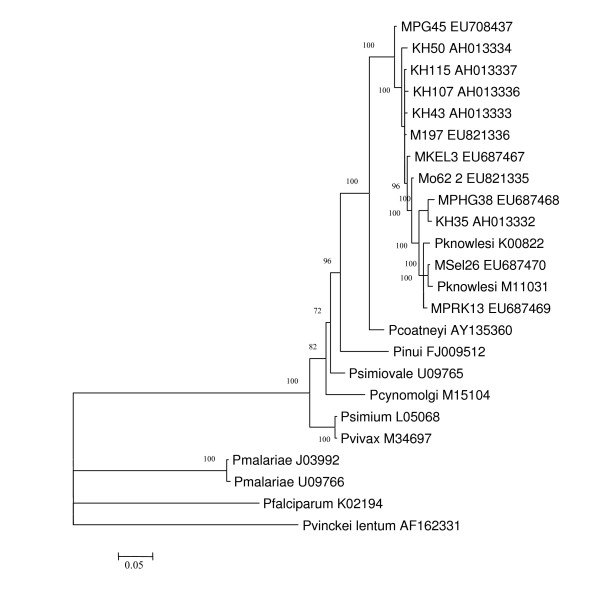
**Phylogenetic tree based on the non-repeat region of the circumsporozoite (*csp*) genes of malaria parasites produced by the Bayesian method.** Figures on the branches are the posterior probabilities from the Bayesian analysis.

The *csp *genes of malaria parasites, from *P. knowlesi-*positive isolates, were successfully amplified, cloned and sequenced. The size of the PCR product ranges from 1050 to1128 bp. Phylogenetic analysis inferred from the NJ method (Figure 2) showed that malaria parasites isolated from these samples clustered with the reference *P. knowlesi *obtained from Gen bank and with those reported by others [3]. When the flanking regions of the *csp *genes, from isolates obtained in this study, were compared with the reference *P. knowlesi *Nuri strain (M11031), the pairwise identity ranges from 97.1% to 99.6% (data not shown). Furthermore, a clone from a mosquito isolate shared an identical sequence with KH115, which was isolated in Sarawak, East Malaysia. The topology obtained using the Bayesian method (Fig 3) is comparable to the NJ tree, which strongly supports the results obtained using the NJ method.

## Discussion

From these preliminary data it is evident that the fifth human malaria parasite *P. knowlesi *is present in most states of peninsular Malaysia. With better molecular diagnostic techniques one can differentiate between *P. knowlesi *and *P. malariae*. The extent of the problem would depend on the cohabitation of humans, non-human primates and the presence of the potential vectors, which are simio-anthropophagic as shown in this study. In a laboratory setting it has been shown that *P. knowlesi *was transmitted by mosquito bites from monkey to monkey, from monkey to humans, from human to human and from human back to monkeys [30]. At that time it was postulated that when human malaria cases reaches a low level, the possibility of reseeding the human population with simian malaria parasites could be highly significant [30]. This perhaps is due to the declining anti-plasmodial immunity in humans leading to increased susceptibility to simian parasites. Most of these cases are occurring in malaria-free areas which are not subject to control activities. There have also been reports of mortality associated with knowlesi malaria infection in humans [6]. This shows that simian malaria could pose a serious problem to public health.

This study has shown that there is a link between *An. cracens*, humans and monkeys. Human cases of *P. knowlesi *are occurring in areas purportedly free from the four human malarias, but where *An. cracens *is found and where monkeys are positive for *P. knowlesi*. A previous study showed that *An. cracens*, an important vector of human malaria, was also positive for *P. inui*, another simian malaria [31]. This mosquito was found feeding on monkeys at the canopy and humans on the ground [31]. In Malaysia, *An. cracens *was first reported in Perlis, the Northern most state bordering Thailand and a later study showed that it was also present in the state of Terengganu [13,29]. This is the first report of the presence *An. cracens *in Kuala Lipis, Pahang and it has now been incriminated as the vector of *P. knowlesi*. Both positive mosquitoes had more than 1,000 sporozoites showing that they are efficient vectors.

*Anopheles maculatus *is the most important vector of human malaria in peninsular Malaysia [28]. It has been shown to be susceptible to simian malaria in the laboratory [32,33] and coming to monkey bait in the canopy [15,25]. However, in our study areas *An. maculatus *has been found only in small numbers biting humans and none in a monkey- bait-trap.

Earlier workers in Malaysia [15] felt strongly that simian malaria will not be easily transmitted to humans. They must have based their conclusions on the fact that in the coastal areas, where monkeys were heavily infected with *P. knowlesi*, the vector *An. hackeri *was highly zoophilic. In the same area, they dissected *An. lesteri *which was found feeding on humans and macaques but were negative by dissection although the macaques were heavily infected. Based on these findings, the chances of humans being infected with simian malaria were deemed remote. In the early days, perhaps the monkeys were not living in close association with humans. Currently with development and deforestation the macaques have come close to human habitation and those in the semi-urban areas were positive for malaria parasites.

Long-tailed macaques are also found in close association with humans in the urban areas but fortunately those found in the urban areas are free of malaria parasites. This could be due to the absence of competent vectors that could transmit malaria parasites.

The rain forests of Southeast Asia occupy hilly areas over parts of Indochina, Thailand, Myanmar, Indonesia and the Philippines and in most of these areas human cases of *P. knowlesi *have been reported [4,5,8]. Natural hosts of *P. knowlesi *such as the long and pig-tailed macaques abound in this region and so does the *An. leucosphyrus *group of mosquitoes. Thus, in the future it will be of no surprise if more human cases of *P. knowlesi *malaria are reported in these areas, contrary to what was perceived by the earlier scientists.

In Sarawak, Malaysian Borneo, *An. latens *has been incriminated as the vector [34] and the biting ratio of monkey to human was 1:1.3 (25). While *An*. *cracens *biting ratio of monkey to humans was 1: 5.6. This shows that *An. cracens *prefer to bite humans compared to monkeys, and could probably explain for fewer cases in peninsular Malaysia. *Anopheles kochi *was found biting monkeys in monkey-baited traps but was not found biting humans. Thus, the possibility is there for it to maintain the infection in the natural hosts.

From the current study, ninety seven percent of the macaques from Kuala Lipis were positive for malaria parasites and the presence of vectors in the area demonstrate that simian malaria in humans may pose to be a public health problem in the near future. Some human cases of P. *knowlesi *in Kuala Lipis were infected in the vicinity of their houses or in the fruit orchards. Sporadic collections of mosquitoes in the case areas revealed the presence of *An. cracens *and macaques were also sighted in those areas. A recent study in Thailand [35] failed to detect *P. knowlesi *in macaque populations in the area where the first case was reported [4].

## Conclusion

This study has established that *An. cracens *is the vector of *P. knowlesi *in Kuala Lipis, Pahang and cases of *P. knowlesi *have occurred in humans in areas where long tailed macaques have also been found infected with *P. knowlesi*. These findings are important for the planning and control of malaria strategies in the future, especially in the elimination of malaria.

## Competing interests

The authors declare that they have no competing interests.

## Authors' contributions

IV, NA, LHS conceived the study, IV, CHT were responsible for the preparation of the manuscript, IV, NAY, AIJ, YY, AAH, NA were responsible for field collection, supervision, identification and processing of mosquitoes and collection of blood from monkeys. IV, NAY, AIJ, CHT were responsible for the molecular work, CHT analysed sequence data. All authors have read and approved the manuscript.
